# Survey data on socioeconomic demographics of drug-free families living in high-risk drug environments in East Coast States of Peninsular Malaysia

**DOI:** 10.1016/j.dib.2024.110454

**Published:** 2024-04-18

**Authors:** Abdul Rahman Abdul Latip, Siti Salina Abdullah, Nor Hayati Sa'at, Nurul Nabila Syaqira Hanizam, Khuzaimah Mustapa, Nur Ameera Rushdi, Engku Nurnajihah Che Ku Muda, Suzaily Wahab

**Affiliations:** a Faculty of Business, Economic and Social Development, Universiti Malaysia Terengganu, 21030 Kuala Nerus, Terengganu, Malaysia; b Centre for Ocean Governance, Institute of Oceanography and Environment, Universiti Malaysia Terengganu, 21030 Kuala Nerus, Terengganu, Malaysia; c Department of Psychiatry, Universiti Kebangsaan Malaysia Medical Centre, 56000 Kuala Lumpur, Malaysia

**Keywords:** Drug, Drug prevention programs, High-risk area, Parent

## Abstract

The dataset represents the socioeconomic demographic of drug-free families living in a high-risk drug environment in East Coast states of Peninsular Malaysia. The purposive sampling technique was used to choose among drug free families living in high drug environment, have children aged between 13 and 17 and none of the family members involved in any substance abuse cases via face-to-face interview questionnaires between 3rd April 2021 and 8th April 2021, gathering a total of 200 responses. The survey assessing socioeconomic demographic information (14 items) and involvement in drug prevention programs (8 items). The survey data was analyzed using descriptive analysis through frequencies and percentages. The data will assist the government, policymakers, and researchers to improve the content and delivery of family-based drug prevention programs and improve family awareness on risk and protective factors towards preventing drug abuse among youths.

Specifications Table

**Table:** 

Subject	Social Studies
Specific subject area	Social Development
Data format	Raw, Analysed, Filtered, Descriptive analysis statistics
Type of data	Tables
Data collection	The data was collected through a face-to-face interview questionnaire conducted with drug-free families in Malaysia using purposive sampling.
Data source location	Region: Asia Country; East Coast States of Peninsular Malaysia
Data accessibility	Repository name: Mendeley Data Data identification number: DOI:10.17632/gvrycbk9kv.1 Direct URL to data: https://data.mendeley.com/datasets/gvrycbk9kv/1 Instructions for accessing these data: Mendeley Data

## 1 Value of the Data

• The data are important because this is the first survey that analyzed the characteristics of drug free families living in high-risk drug environments in east coast states of Peninsular Malaysia.
• The data represents the involvement of families in drug prevention programs organized by various agencies in Malaysia.
• The data will be useful for researchers who want to compare with similar studies related to families and drug-related studies.
• The dataset will help stimulate in-depth research on parents' risk and protective factors in preventing their family members from engaging in any substance abuse activities.
• The data contributes important information for the government, policymakers, and researchers to improve the content and delivery of family-based drug prevention programs.
• The objective of this survey data is to provide meaningful information regarding the socioeconomic demographic of drug free parents living in high risk drug environment and their involvement in drug prevention programs.

## 2 Background

The objective of this survey data is to provide meaningful information regarding the socioeconomic demographic of drug free parents living in high risk drug environment and their involvement in drug prevention programs.

## 3 Data Description

The data set provides insightful information based on a survey related to the socioeconomic demographics of drug-free parents living in high-risk environments and their involvement in drug prevention programs. The survey involved 200 families living in high-risk drug environments located in East Coast states of Peninsular Malaysia. The data includes a significant group of variables (A) socioeconomic demographic including; gender, age, race, marital status, place of current residents, number of children, number of children in primary school, number of children in secondary school, number of working children, number of children with disabilities, number of household members, level of education and employment categories, (B) parents involvement in drug prevention programs organised by various agencies including National Anti-Drug Agency, Ministry of Education, Ministry of Youth and Sport, The National Population and Family Development Board (LPPKN), Department of Information (Ministry of Communications and Multimedia), Department of National Unity and National Integration (Ministry of National Unity) and Non-Government Organisation (NGOs) [1], parents’ interest towards drug prevention program and reasons for involvement [[2], [3]]. Each question regarding parent involvement is rated on a nominal scale in such a way that scores are given for ‘Yes’ for parents who involved in drug prevention programs and ‘No’ for parents who never involved in any drug prevention programs. Further questions regarding the reasons for parents involved in drug prevention programs are rated by seeking new information, increasing knowledge, and availability while reasons for parents who never got involved in drug prevention programs are rated by commitment and health condition (Table 1).

**Table 1:** Socioeconomic demographic characteristics of the participants (*n* = 200).

Variable	Freq (n)	%
**Gender**		
Male	168	84.0
Female	32	16.0
**Age**		
<40	24	12.0
41–59	141	70.5
> 60	35	17.5
**Race**		
Malay	200	100.0
**Marital status**		
Married	169	84.5
Single father	1	0.5
Single Mother	30	15.0
**Area**		
Urban	40	20.0
Rural	160	80.0
**Number of children**		
1–3 person	66	33.0
4–6 person	101	50.5
7–10 person	31	15.5
Above 11 persons	2	1.0
**Number of children in primary school**		
None	112	56.0
1–3 person	88	44.0
**Number of children in secondary school.**		
1–3 person	199	99.5
4–6 person	1	0.5
**Number of working children**.		
None	102	51.0
1–3 person	67	33.5
4–6 person	24	12.0
7–10 person	7	3.5
**Number of children with disabilities.**		
None	196	98.0
1–3 person	4	2.0
**Number of household members**		
1–3 person	22	11.0
4–6 person	124	62.0
7–10 person	53	26.5
> 11 persons	1	0.5
**Level of education**		
No formal education	2	1.0
Primary school	15	7.5
Secondary lower (SRP/PMR)	37	18.5
Secondary upper (SPM)	120	60.0
Certificate/STPM/Diploma	14	7.0
Bachelor/Master and above	12	6.0
**Types of respondent's career**		
Skilled	24	12.0
Semi-skilled	104	52.0
Low-skilled	72	36.0
**Head of household's income (monthly)**		
>RM1000	91	45.5
RM1001-RM2000	67	33.5
RM2001-RM3000	21	10.5
RM3001-RM4000	5	2.5
>RM4001	16	8.0

## 4 Experimental Design, Materials and Methods

This research adopted a cross-sectional survey method to determine the socioeconomic demographics of drug-free parents living in a high-risk drug environment and their participation in drug prevention programs.

The study locations were chosen based on specific high-risk areas with high crime rates, social problems, and a high number of drug addicts, as identified by the authorized drug agency. In addition, since the study sample was selected using purposive sampling, the researcher directly consulted with local authorities to ensure they met the study's requirement, namely, that none of family members were currently involved or had a history of involvement with drug addiction. The data set contains 200 responses from the head of the household that were collected via face-to-face interviews from 3rd to 8th April 2021. Purposive technique was used to select the respondents. The inclusion criteria were; (i) drug free family lives in high-risk drug environment (high crime rates, social problems, and a high number of drug addicts) (ii) family with teenagers aged between 13 and 17 (iii) none of the family members involved in any drug activities. A total of 210 responses were received, but 10 responses were eliminated due to not met the criteria. Finally, 200 responses were used for further analysis. The data on drug prevention and intervention programs conducted by agencies was collected from the National Anti-Drug Agency website. The data were analyzed using descriptive analysis via frequencies and percentages (Tables 2 and 3).

**Table 2:** Respondent's involvement in drug prevention programs in Malaysia.

Program	Activity	Yes		No	
		Freq (n) %		Freq (n) %	
Focused Drug Prevention Program (National Anti Drug Agency)	Drug-Free Family (Family on Aler*t*)	6	3.0	194	97.0
	Drug-Free Workplace	7	3.5	193	96.5
	Drug-Free Community	5	2.5	195	97.5
	Drug-Free Educational Institutions	3	1.5	197	98.5
	Anti Drugs Squad	6	3.0	194	97.0
	Public Awareness and Anti-Drug program	4	2.0	196	98.0

Prevention and Intervention Education Program (Ministry of Education Malaysia)	*Waja Diri*	–	–	200	100.0
	National Child Intervention Program - *Program Intervensi Anak Negara* (INTAN)	–	–	200	100.0
	Improve Knowledge Determine Active Program - *Program Ilmu Maju Aktif Nekad* (IMAN)	–	–	200	100.0

Youth Prevention Program (Ministry of Youth & Sport Malaysia)	The More You Use, The Less You Live Program (campaign, concert, outreach, seminar and follow-up session)	–		200	100.0

Parenting Seminar (Lembaga Penduduk dan Pembangunan Keluarga Negara)	Prevention education and parenting skills program	7	3.5	193	96.5

Prevention and Intervention Education Program (Department of Information)	1Malaysia Community Info	–	–	200	100.0
	*Sepakat*	–	–	200	100.0

Prevention Education Program with the Department of National Unity and Integration	*Sayangi Komuniti*	–	–	200	100.0
	Volantary Patrol Scheme + omnipresence (AADK)	1	0.5	199	99.5
	Training & Development of Officers	–	–	200	100.0
	Training for unity kindergarten teachers	–	–	200	100.0

Prevention Education Program with NGOs	Youth, Adolescent, Workplace, Community Prevention Education	–	–	200	100.0

**Table 3:** Interest in drug prevention program.

Reason	Yes (*n*)	%	No (*n*)	%	
New info on drug	34	17	–	–	
Increase knowledge on drug prevention	65	32.5	–	–	
Availability		5	2.5	–	–
Commitment		–	–	73	36.5
No reasons		–	–	7	3.5
Health condition		-	–	16	8

## Limitations

The data from respondents contains more males than females due to the focus of this study on the head of the family.

## Ethics Statement

The research was approved by the ethics and research review committee at Universiti Malaysia Terengganu, Malaysia (UMT/JKEPM/2022/106). The informed consent was obtained during conducting the data collection.

## CRediT authorship contribution statement

**Abdul Rahman Abdul Latip:** Writing – original draft, Writing – review & editing. **Siti Salina Abdullah:** Writing – original draft, Conceptualization, Formal analysis, Writing – review & editing. **Nor Hayati Sa'at:** Methodology, Software, Formal analysis. **Nurul Nabila Syaqira Hanizam:** Formal analysis, Software. **Khuzaimah Mustapa:** Formal analysis, Software. **Nur Ameera Rushdi:** Resources. **Engku Nurnajihah Che Ku Muda:** Resources. **Suzaily Wahab:** Writing – review & editing.

## Data Availability

DATA AND QUESTIONNAIRE (MALAY & ENGLISH VERSION) (Original data) (Mendeley Data).
